# Novel probiotic treatment of autism spectrum disorder associated social behavioral symptoms in two rodent models

**DOI:** 10.1038/s41598-022-09350-2

**Published:** 2022-03-30

**Authors:** Kitti Mintál, Attila Tóth, Edina Hormay, Anita Kovács, Kristóf László, Anita Bufa, Tamás Marosvölgyi, Béla Kocsis, Adorján Varga, Zoltán Vizvári, Renáta Cserjési, László Péczely, Tamás Ollmann, László Lénárd, Zoltán Karádi

**Affiliations:** 1 Institute of Physiology, Medical School, University of Pécs, Pécs, Hungary; 2 Cellular Bioimpedance Research Group, Szentágothai Research Centre, University of Pécs, Pécs, Hungary; 3 Institute of Bioanalysis, Medical School, University of Pécs, Pécs, Hungary; 4 Department of Medical Microbiology and Immunology, Medical School, University of Pécs, Pécs, Hungary; 5 Department of Environmental Engineering, Faculty of Engineering and Information Technology, University of Pécs, Pécs, Hungary; 6 Institute of Psychology, ELTE Eötvös Loránd University, Budapest, Hungary

**Keywords:** Drug discovery, Neuroscience, Medical research

## Abstract

The prevalence of autism spectrum disorder (ASD) has rapidly increased in the past decades, and several studies report about the escalating use of antibiotics and the consequent disruption of the gastrointestinal microbiome leading to the development of neurobehavioral symptoms resembling to those of ASD. The primary purpose of this study was to investigate whether depletion of the gastrointestinal microbiome via antibiotics treatment could induce ASD-like behavioral symptoms in adulthood. To reliably evaluate that, validated valproic acid (VPA) ASD animal model was introduced. At last, we intended to demonstrate the assessed potential benefits of a probiotic mixture (PM) developed by our research team. Male Wistar rats were used to create antibiotics treated; antibiotics and PM treated; PM treated, VPA treated; VPA and PM treated; and control groups. In all investigations we focused on social behavioral disturbances. Antibiotics-induced microbiome alterations during adulthood triggered severe deficits in social behavior similar to those observed in the VPA model. Furthermore, it is highlighted that our PM proved to attenuate both the antibiotics- and the VPA-generated antisocial behavioral symptoms. The present findings underline potential capacity of our PM to improve social behavioral alterations thus, indicate its promising therapeutic power to attenuate the social-affective disturbances of ASD.

## Introduction

Autism spectrum disorder (ASD) is a complex neurodevelopmental disorder characterized by impaired social interactions and communication along with the presence of disturbances of repetitive stereotyped behaviors^1,2^. Eventually, difficulties with social interactions have been supposed to be the major deficits and these most severe symptoms of the disorder persist across the lifespan^3–6^. Therefore, one of the key elements of the therapeutic approaches is the development and evaluation of interventions to strengthen the social skills of the patients^6^. The current solutions are limited to social skills training techniques and programs or using human-like robot technologies^7,8^. However, despite all these strategies, there are no recognized pharmacological treatments to cure the core features of ASD. Nevertheless, there are studies where sulforaphane, oxytocin or arginine-vasopressin treatments were able to improve the general condition of patients^9–11^. Along with these developments valid animal models are required to test the new therapeutic strategies aiming to improve the core features of ASD. These models also proved to be useful to examine the morphological effects of specific causal factors on characteristic neuropathology of the condition^12^. There are two main types of animal models for modelling the pathological features of ASD: genetically- and environmentally induced animal models^13,14^. The use of genetic manipulations could be helpful to elucidate mechanisms related to the genetic etiology and the pathogenesis of ASD. Animal models for ASD induced by environmental factors (include prenatal infection, valproic acid, propionic acid, poly (I:C) or lipopolysaccharides exposure) can result in a behavioral phenotype reminiscent of ASD, with specific impairments in social interaction, communication and repetitive behaviors^13–17^. The valproic acid (VPA) animal model is one of the most frequently studied rodent models of ASD in which a single prenatal exposure to VPA in rodents results in lifelong behavioral disturbances and differentially alters morphological parameters of hippocampal regions^18–21^. This ASD animal model appears to possess all elements of a relevant and capable model, namely, face, construct, and predictive validity, and, therefore, it can represent an important strategic tool for developing novel therapeutic approaches^18,22,23^.

Epidemiological studies have reported a gradual increase of the prevalence of ASD in the last decades^24^. This continuous rise is suggested to appear due to the increasing contamination by environmental factors such as toxins, heavy metals, chemicals, maternal infection, various pathogens, and, recently the escalating use of antibiotics has also been proposed as a causal factor^25–29^. Investigations over the last few years suggested that broad-spectrum antibiotics exposure slightly increases the risk of autism in early childhood^30,31^, however, the currently available research data pool does not substantiate the notion that either the pre- or post-natal antibiotics exposure is really one of the risk factors for ASD^27^. Nevertheless, broad-spectrum antibiotics treatments in animal studies can provide another useful way to examine the relationship between the effects of environmental factors, such as antibiotics exposure, and the multiple symptomatology of ASD^27^. Furthermore, it has also been demonstrated that the antibiotics induced manipulation of complex composition of the intestinal microbiota can ultimately affect brain functions leading to behavioral alterations, through the microbiome–gut–brain axis^27,31–33^. Certain antibiotics, their definite dosages, during assigned perinatal stages appear to affect social behavior^33^. In adulthood, the effect of antibiotics treatment on social behavior is less studied, but still there are some investigations that provided evidence for that the usage of antibiotics can elicit behavioral changes^33,34^. The employment of probiotics and prebiotics also modulates the composition of the gastrointestinal (GI) microbiome, and recently, probiotics are not only utilized to just reduce unwanted aftereffects following the antibiotics treatments^35^, but some beneficial microbe types are also applied to improve several peripherally and/or centrally controlled functions. It is worth noting that such medications are successfully used to improve symptoms of several neurobiologically determined disorder-like conditions, psychiatric disorders, depression, etc.^36,37^. For instance *Lactobacillus*- and *Bifidobacterium* species, which are part of the native microbiota, have beneficial health properties as they could reduce oxidative stress and possess anti-inflammatory characteristics, further they are able to repair gastrointestinal barrier functions, manage to modify several neurotransmitters- and cytokines levels and they could modulate various signaling pathways^38–42^. Furthermore, short chain fatty acids (SCFAs), the main microbiome metabolites, produced by bacterial fermentation, can directly or indirectly affect microbiome–gut–brain axis and their dose might be critical in determining the effects on behavioral and psychophysiological processes^43^. For all that, the use of antibiotics and distinct probiotics with well-defined characteristics can modulate the gut microbiota, thus, provide us a tool to examine the relationship between ASD associated symptoms and the functioning of microbiome–gut–brain axis.

The present study aimed to investigate whether chronic antibiotics treatment in adult rats could cause social behavioral abnormalities similar to those described in the ASD. Accordingly, we adapted the VPA animal model of ASD to evaluate and compare this disorder associated characteristic social behavioral phenomena in adulthood. Another further goal of this study was to treat the rats having antibiotics-induced behavioral abnormalities by a probiotic mixture (PM) designed by our research team. Our assumption was that the PM could reduce the pathological behavioral patterns through the beneficial changes induced in the GI microbiome which could be a possible new therapeutic approach to cure social behavioral symptoms.

## Methods

### Animals

In the present study, in total, 60 male Wistar laboratory rats (antibiotics treated groups 40, valproic acid treated groups 20) were used (10 weeks old at start of treatments). All animal experiments were conducted according to federal and local ethical guidelines, and the protocols were approved by the National Scientific Ethical Committee on Animal Experimentation of Hungary (BA02/2000–15/2020 and BA02/2000–16/2020, Pécs University, Medical School; Hungarian Government Decree, 40/2013. (II. 14.); NIH Guidelines, 1997; European Community Council Directive 86/609/EEC 1986, 2006; European Directive 2010/63/EU of the European Parliament). The present study is reported in accordance with ARRIVE guidelines. The animals were kept individually in a light and temperature-controlled room (12:12 h light–dark cycle; 21 ± 2 °C; humidity 55–60%). To exclude sex as an additional independent variable, only male rats were used in this study. All experimental groups received ad libitum the same laboratory food pellets (LT/R standard rodent food pellet, Innovo Kft, Isaszeg, Hungary) and tap water.

### Treatments of the antibiotics groups

**Figure 1:**
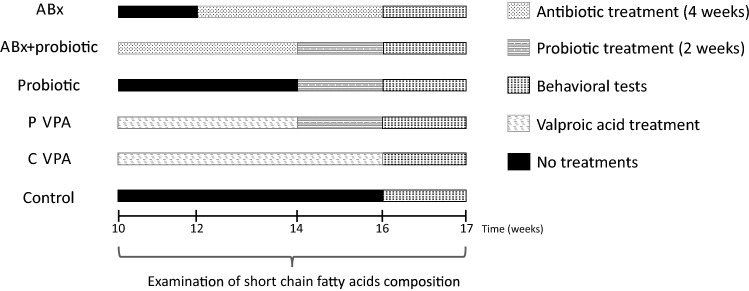
Experimental arrangement of the treatments. Experimental treatments for the groups including demonstration of time and duration of antibiotics and probiotic treatments and those of behavioral testing. ABx: broad-spectrum antibiotics treated group, ABx + probiotic: broad-spectrum antibiotics- and probiotic treated group, Probiotic: probiotic treated group, P-VPA: valproic acid and probiotic treated group, C-VPA: valproic acid treated group, Control: control group without any treatment.

Animals have been divided randomly (one or two rats/ litter/group) into four groups: 1. Control group (control); 2. Antibiotics treated group (ABx); 3. Antibiotics and probiotic treated group (ABx + probiotic); 4. Probiotic treated group (probiotic) (Fig. 1). To effectively deplete the gut microbiota the rats of the antibiotics treated groups received broad-spectrum antibiotics mixture for 4 weeks in adult animals (10 weeks old at the start of treatment). The antibiotics cocktail was dissolved in their drinking water to avoid any chronic stress-induced adverse effects. The antibiotics mixture was chosen based on published protocols^34^, consisted of ampicillin (1 g/l), vancomycin (500 mg/l), ciprofloxacin HCl (20 mg/l), imipenem (250 mg/l) and metronidazole (1 g/l). This antibiotics cocktail was replaced by freshly made cocktail every 3 days. Probiotic treated and control animals received tap water in the absence of any antibiotics which was also changed every 3 days. (Pilot experiment was conducted (n = 8) to exclude possible behavior changes due to antibiotics-induced intestinal discomfort or pain, detailed in the Supplementary Information; Supplementary Table S1) After this antibiotics exposure in the ABx + probiotic group, and the probiotic group were given our PM of specified cfu/d (colony forming units/day), oral gavage every day for 2 weeks. Throughout the whole experiment water and food consumption were measured every day and the animals’ weights were measured every 3 days. Fresh faecal pellets were collected every week for monitoring the alterations of the SCFAs. Behavioral tests started after the treatments.

### Treatment with the probiotic mixture

Our specific PM contained four beneficial bacterial species (*Lactobacillus* spp., *Bifidobacterium* spp.) and this PM is a know-how under the license of the University of Pécs. The origin of the strains was Leibnitz, institut DSMZ, Deutsche Sammlung von Mikroorganismen und Zellkulturen GmbH Deutschland. After we received the strains in freeze—dried form we started to cultivate them following the protocol sent by the DSMZ firm. After successful cultivation we checked the strains with different biochemical methods among them MALDI-TOF. After identification we stocked the strains in glycerol preservative fluid at − 80 °C. Before cultivation for experiments we reactivated the strains from − 80 °C on optimal media at presence or absence oxygen depending on whether the bacterium was aerobic or anaerobic. Lactobacillus spp. strains were cultivated in 100–100 mL liquid Rogosa medium (OXOID Ltd. UK) for 2 nights at 37 °C in a shaker incubator at 200 rpm. The Bifidobacterium spp. were cultivated on fastidious anaerobic agar CE plates and broth (Neogen Europe Ltd. UK). First, the strains were cultivated on plates. Anaerobic conditions were produced in anaerobic jar with GEnbag anaer (BioMérieux SA France). After 2 days cultivation the colonies from plate were inoculated into 100–100 mL anaerobic broth. After 2 days cultivation both the aerobic (for Lactobacillus) and anaerobic (for Bifidobacterium) fluids were centrifuged at 4 °C with 5000 rpm for 5 min. The sediments were resuspended in physiological saline solution (0.9% sodium chloride). We mixed the suspensions of Lactobacillus and Bifidobacterium strains and filled with physiological saline solution till 10 mL final volume. This 10 mL mixture was used in experiment for 10 mice. This mixture was produced from day to day and used immediately. From time to time we checked the sediment before we mixed them for the number and identity of bacteria. In this checking process we used double dilution method and MALD-TOF.

### Treatments of the valproic acid groups

The valproic acid animal model has been generated as previously described^44^. Male and female Wistar rats were mated one overnight, and the morning when a vaginal plug was found was designated as the first day of gestation. Pregnant female rats received a single intraperitoneal injection of 500 mg/bwkg valproate (Sigma-Aldrich; P4543) dissolved in physiological saline at a concentration of 250 mg/mL on the 12.5th day of gestation, and control females were injected with physiological saline at the same time. Valproate-treated females were healthy individuals and the number of animals per litter was approximately 25% lower in VPA compared to control dams. Females were housed individually and were allowed to raise their own litters. Male offsprings, when reached 8 weeks, for the groups separated and randomly selected from each litter and they were kept individually throughout the experiment. These animals have been divided randomly (one or two rats/ litter/group) into two groups: probiotic treated valproate group (P-VPA); 2. control valproate group (C-VPA) (Fig. 1). The P-VPA group were given the above described PM every day for 2 weeks. Water and food consumption were measured every day and the animals’ weights were measured every 3 days during the experimental period. Fresh faecal pellets were collected every week. Behavioral tests were conducted in the same sequence in every group of animals. All 60 animals (n = 10/group) completed each behavioral test and their accompanying tissue samples were used for further analyses.

### Three chambered social interaction test

A three chambered social interaction task was used to assess social behavior. The protocol was adapted from a previously published article^45^. The apparatus (150 × 40 × 40 cm) was divided into three chambers: the nonsocial zone (60 × 40 cm), the social zone (60 × 40 cm) and the centre (30 × 40 cm).The non-social and the social zone contained small circular wire cages with a diameter of 18 cm. The testing arena was cleaned with 1% Incidin after each testing trials. Before the sociability task experimental rats immediately were given a habituation session. They were placed into the centre of the apparatus, where they were allowed to explore it for 10 min. Right away this habituation session test animals were locked in the centre zone for 3 min, while a stranger rat of the same strain and sex was placed into one of the two rat cages in the side chambers. Therefore, one of the side chambers which contained a stranger rat would be the social zone and the other chamber, where the cage remained empty would be the non-social zone. Test animals had no previous interaction with stranger animals for this task and the placement of the stranger rat was randomized and counterbalanced. Following this 3 min centre zone locking, animals were provided 10 min to freely explore the whole apparatus. The entire experimental period was recorded and analyzed by recorded camera shots processed by Noldus EthoVison System (Noldus Information Technology, The Netherlands). We measured the total distance moved, time spent in the side chambers, and during the sociability task latencies to first entry to the side chambers were also determined. Furthermore, direct interactions with either the stranger rat- or empty cages were counted. All types of exploratory behavior were noted, and the sociability index (time spent in the social zone − time spent in the non-social zone)/(time spent in the social zone + time spent in the non-social zone) was also used to indicate a preference to interact with or avoid the stranger rat.

### Short chain fatty acids analysis

We used the protocol of Wall et al.^46^ with modification on the internal standard to measure SCFAs. 100 mg of faecal samples were weighted out and vortex-mixed with 1 ml of distilled water. After standing for 10 min at room temperature, 100 µl (2 mmol/l) Heptanoic acid (Sigma-Aldrich; 43858) as an internal standard was added to them and the samples were centrifuged at 10,000 rpm for 5 min. Then supernatant fluids were collected and filtered before being transferred to the vials. Standard solutions containing 10 mmol/l, 7.5 mmol/l, 5 mmol/l, 2.5 mmol/l and 0.5 mmol/l of acetic acid (Sigma-Aldrich; A6283), propionic acid (Sigma-Aldrich; 94425) and butyric acid (Sigma-Aldrich; 19215), respectively, were used for calibration. The concentration of SCFAs analyses were carried out on an Agilent 6890 N gas chromatograph with a 5975 mass spectrometer detector (Agilent, Santa Clara, CA, USA) fitted with a HP-INNOWAX column (30 m × 0.25 mm × 0.25 µm; Agilent). Helium was used as the carrier gas at a flow rate of 1.5 ml/min. The initial oven temperature was 80 ℃ held for 1 min and ramped up to 200 °C at 20 °C/min and held for 2 min. The injection port was adjusted at 250 °C and split injection mode was used, the injection ratio was 20:1. The injection volume was 1 µl. The interface temperature was maintained at 250 °C, the source temperature and the quadrupole mass analyzer temperature were set at 230 °C and 150 °C. The solvent delay was 3 min. The mass spectrometer was operated at 70 eV in the electron impact (EI) mode and the scanned mass range was 50–300 amu.

### Histology

At the end of behavioral experiments animals were euthanized and transcardially perfused with physiological saline followed by 4% formalin solution. The brains of 6 animals/groups were fixed in 4% formalin, afterwards they were cut into 40 µm sections containing the hippocampus. The sections were stained using Cresyl Violet staining. Briefly, they were immersed in 70 and 50% ethanol and double distilled water for 5 min. The slides were then stained for 7 min in 0.5% cresyl violet solution, and then briefly rinsed in distilled water. They were then dehydrated in 70% ethanol with 10 drops of 100% acetic acid, 70, 90, double 90% ethanol, double 1-propanol for 1 min each. The slices were placed in 1:1 mixed 1-propanol and xylene for 5 min and then in xylene for overnight and then coversliped. The open-source image-processing software package ImageJ (NIH) was used for image analysis. The diameter of the hippocampal regions (subiculum, CA1, CA2, CA3, dentate gyrus) of the selected section (bregma −3.6 to 3.8 mm) were measured.

### Statistics

In the three chamber social interaction test the stranger- and empty cage latency, interactions with the stranger rat and the social index did not pass through normality- and homogeneity test, in these cases non-parametric Kruskal–Wallis test was used to analyze group differences between C-VPA, P-VPA, control, ABx, ABx + probiotic and probiotic treated groups, and if a difference was found to be significant, pair-wise comparison was done using the Mann–Whitney *U*-test. Data were presented as median (interquartile range [IQR]). For the other comparisons in the three chamber social interaction test, body weight, food- and water consumptions among the groups parametric one-way ANOVA was used and Post-hoc group mean comparisons were conducted using Tukey’s post hoc test. All of these data were presented as mean ± standard error of the mean (SEM). The analysis of the SCFAs within the groups was completed by Friedman test, and to assess differences in before- and after treatment outcomes between groups were used Kruskal–Wallis test, the results were demonstrated as median (IQR). The data of histological measurements did not have normal distribution, therefore, Kruskal–Wallis test and Mann–Whitney *U*-test were conducted. To remove the influence of litter effects we applied one animal per litter to the groups, although there were some situations where more than one animal per litter was used, in those circumstances their values were averaged^47^. Significance was denoted with selection of a *p* value of < 0.05. Statistical analyses were conducted using the statistical software package (IBM SPSS Statistics 22).

## Results

### Body weight comparisons, food and water consumptions

Prenatal valproic acid treatment, antibiotics and/or probiotic administration had no significant effect on body weight change compared to the control group (Supplementary Fig. S1). Some of the animals receiving antibiotics had significantly decreased food- and water consumptions during the first period of antibiotics treatment, however, after this first one week period, food and water intakes of the animals were normalized and the treatments did not affect the consumptions (Supplementary Fig. S1).

### Effects of antibiotics- or valproic acid treatment on social behavior

Latency to the first exploration of both the stranger rat and empty cage were analyzed. Although the treatment effects on latency to explore the social zone were non-significant, probiotic treated rats show a tendency to explore later the zone of the empty cage (Table 1).

**Table 1:** Three chamber social interaction test.

3 Chamber social interaction test	ABx n = 8	ABx + probiotic n = 8	Probiotic n = 8	P-VPA n = 7	C-VPA n = 7	Control n = 8
Stranger cage latency (s)	23.00 (10.38–154.13)	19.00 (8.50–77.25)	16.00 (4.88–45.25)	7.00 (5.00–14.75)	40.50 (2.00–103.00)	17.00 (4.75–27.75)
Empty cage latency (s)	4.75 (3.00–13.50)	19.50 (2.00–70.88)	150.25 (68.13–294.00)	46.00 (4.50–217.00)	51.00 (18.50–60.00)	36.00 (4.88–86.88)
Sociability index	-0.48 (-0.63–0.10) ^abcd^	0.74 (0.54–0.88)^ae^	0.45 (0.31–0.63)^bf^	0.79 (0.51–0.81)^cg^	-0.19 (-0.47–0.24)^efgh^	0.69 (0.26–0.75)^dh^
Social zone exploration frequency	24.50 ± 5.74	55.88 ± 9.37	24.81 ± 6.98	43.71 ± 9.73	30.79 ± 8.55	40.44 ± 6.91
Non-social zone exploration frequency	39.94 ± 11.36	17.13 ± 4.10	20.44 ± 6.99	12.21 ± 3.18	27.21 ± 6.22	18.19 ± 3.41
Total distance moved (cm)	2104.82 ± 268.13	2536.27 ± 156.76	2856.05 ± 637.81	2134.07 ± 165.78	2077.91 ± 214.07	2839.09 ± 273.11
No. of rearing behavior	13.38 ± 2.60	17.06 ± 3.02	15.38 ± 3.02	18.57 ± 3.59	15.14 ± 3.98	19.31 ± 3.07
No. of grooming behavior	5.94 ± 1.44	6.13 ± 1.13	4.25 ± 0.94	8.07 ± 2.62	8.50 ± 1.46	8.88 ± 1.72

ABx: broad-spectrum antibiotics treated group, ABx + probiotic: broad-spectrum antibiotics- and probiotic treated group, Probiotic: probiotic treated group, P-VPA: valproic acid and probiotic treated group, C-VPA: valproic acid treated group, Control: control group. Values of the stranger cage latency, the empty cage latency and sociability index are median (IQR) and the social- and non-social zone exploration frequency, total distance moved values, number of raring behavior and number of grooming behavior are means and SEMs. One-way ANOVA, Kruskal–Wallis test and Mann–Whitney U-test. Between the groups significances (*p* < 0.05) are represented by distinct (a-h) lowercase letters.

**Figure 2:**
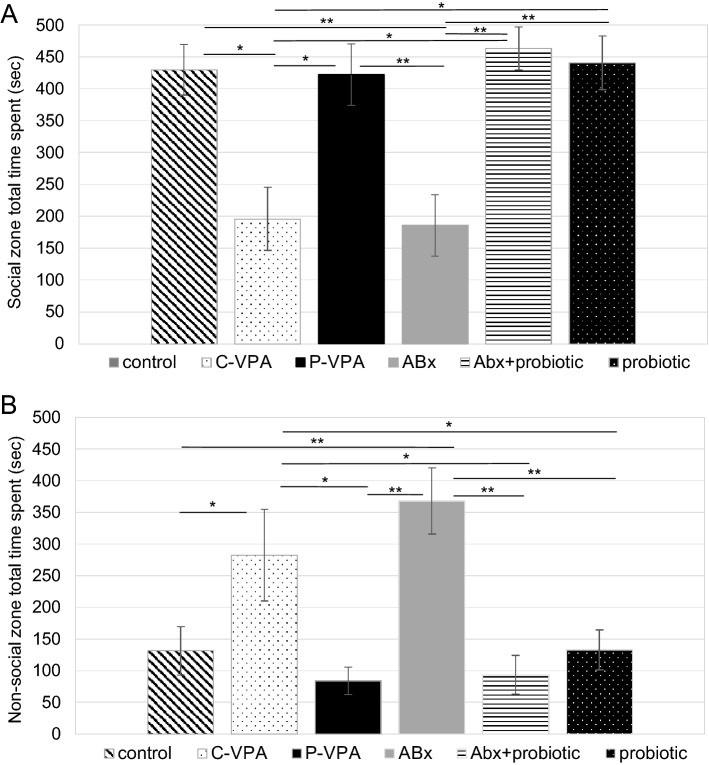
Time spent with the social- (**A**) and non-social (**B**) zone exploration (s) in the three chamber social interaction test. Control: control group; C-VPA: valproic acid treated group; P-VPA: valproic acid and probiotic treated group; ABx: broad-spectrum antibiotics treated group, ABx + probiotic: broad-spectrum antibiotics- and probiotic treated group, probiotic: probiotic treated group. One-way ANOVA (*p < 0.05;**p < 0.01). Data graphed as mean ± SEM.

The total time spent exploring the stranger rat or empty cage was also analyzed (Fig. 2). C-VPA animals spent significantly less time in the social zone (*p* = 0.013) and more time in the non-social zone (*p* = 0.046). Similar to this group, antibiotics treated animals exploring either the stranger (*p* = 0.006) or empty cage (*p* = 0.005) were significantly different from members of the other groups. While compared to the habituation session each group spent balanced time in the social- and in the non-social zone (data not shown).

The sociability index was also estimated to indicate a preference to the stranger rat. This index for the ABx and the C-VPA rats was significantly lower than for the animals of the other groups (*p* = 0.022): it reduced by approximately 70%, compared to the other groups (Table 1).

**Figure 3:**
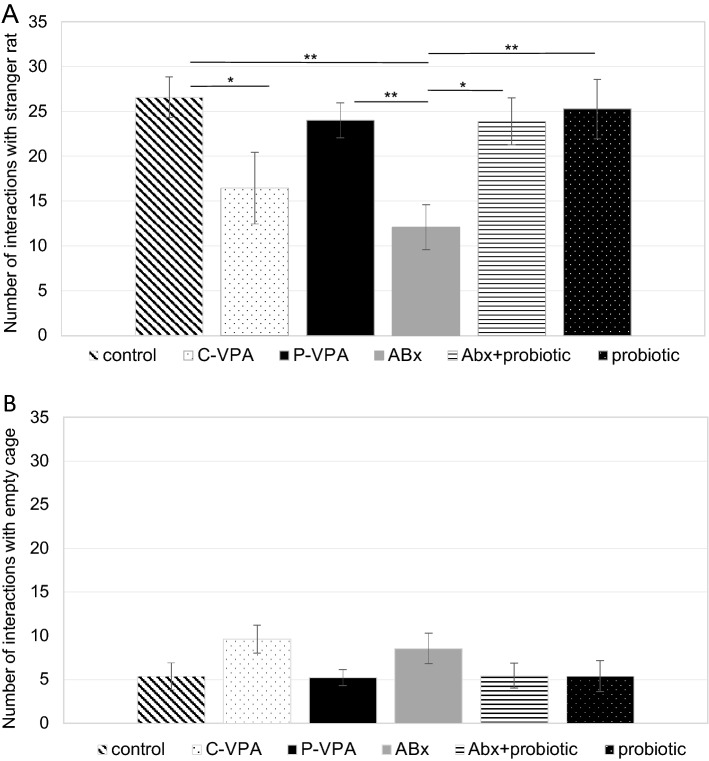
Number of interactions with the stranger rat (**A**) and empty cage (**B**) in the three chamber social interaction test; Control: control group; C-VPA: valproic acid treated group; P-VPA: valproic acid and probiotic treated group; ABx: broad-spectrum antibiotics treated group, ABx + probiotic: broad-spectrum antibiotics- and probiotic treated group, probiotic: probiotic treated group. One-way ANOVA, Kruskal–Wallis test (*p < 0.05;**p < 0.01). Data graphed as mean ± SEM.

Analysis of the exploration frequency in the social- or non-social zone of the groups did not show significant differences (Table 1). Interactions with stranger animal and empty cage were overall calculated (Fig. 3). We did not find any difference among the groups in the empty cage interaction, however, compared to individuals of the control groups, significantly less interactions were observed with the stranger rat in the ABx (*p* = 0.002) and C-VPA animals (*p* = 0.050), moreover, ABx rats differed from the ABx + probiotic (*p* = 0.025), probiotic (*p* = 0.003) and P-VPA (*p* = 0.010) rats, too.

The total distance travelled did not show significant differences among the groups (Table 1). Furthermore, we also evaluated calculated stereotype behaviors, the results showing no significant differences in the examined rearing and grooming behaviors (Table 1).

### Concentration of the short chain fatty acids in relation to the treatments

**Figure 4:**
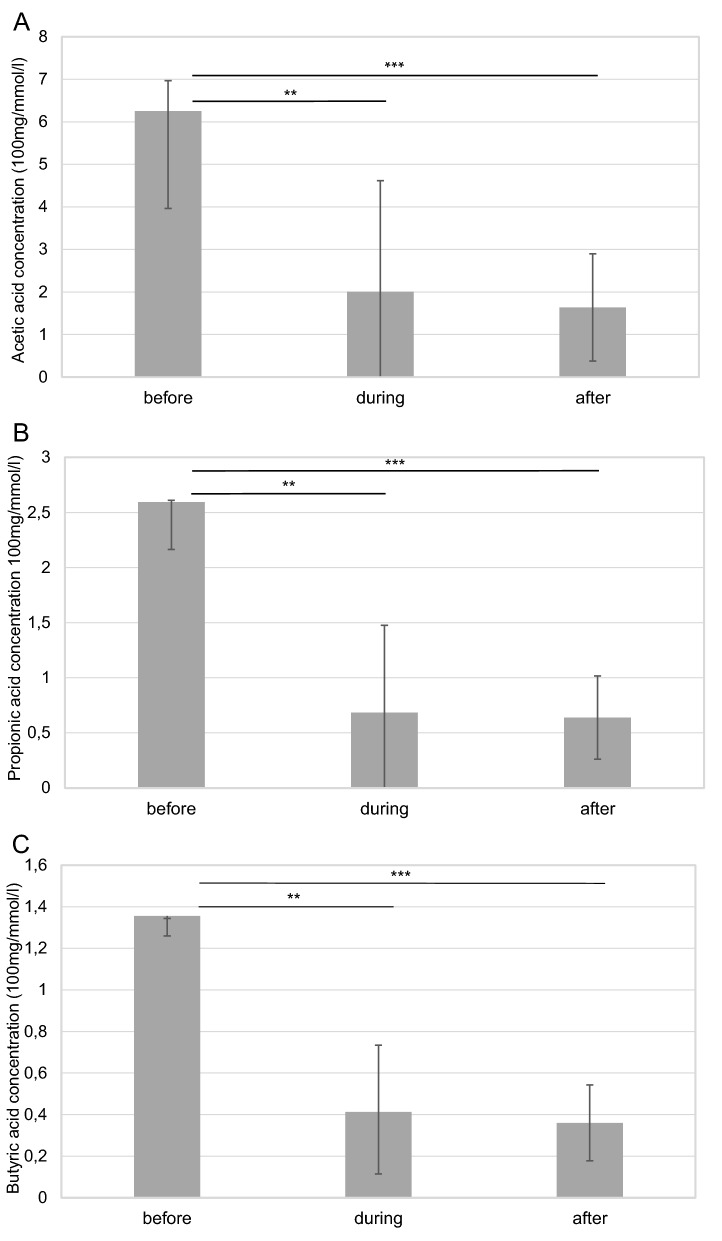
The effect of antibiotics treatments on the acetic acid (**A**), propionic acid (**B**) and butyric acid (**C**) concentrations (100 mg/mmol/l) in the faecal samples before-, during- and after the antibiotics treatment. Abx: broad-spectrum antibiotics treated group; Friedman test (**p < 0.01; ***p < 0.001). Data graphed as median ± IQR.

**Figure 5:**
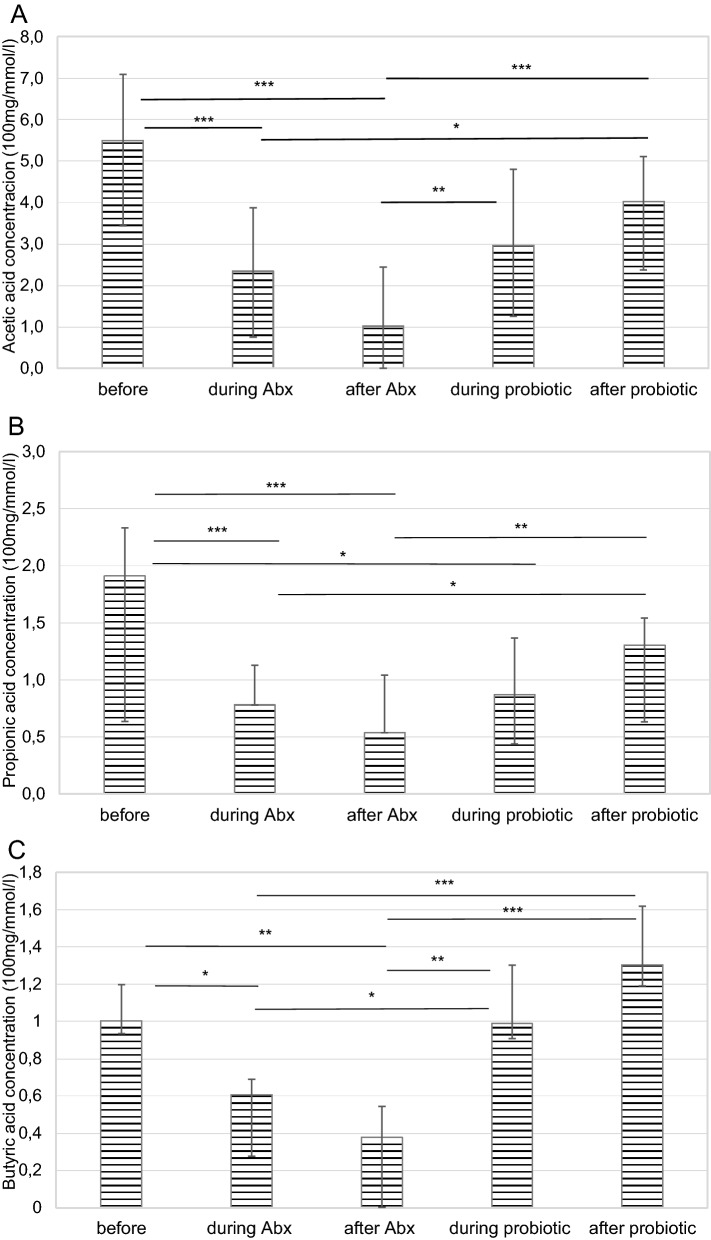
The effect of antibiotics and probiotic treatments on the acetic acid (**A**), propionic acid (**B**) and butyric acid (**C**) concentrations (100 mg/mmol/l) in the faecal samples before the treatments, during- and after the antibiotics treatment, as well as during- and after the probiotic treatment. Abx + probiotic: broad-spectrum antibiotics treated and after probiotic treated group; Friedman test (*p < 0.05; **p < 0.01; ***p < 0.001). Data graphed as median ± IQR.

Concentrations of the SCFAs were analyzed in all groups before, during and after the treatments. Median (IQR) values of the concentrations of the SCFAs are given in Table 2. Significant differences were observed in all the three measured fatty acid concentration after the antibiotics treatment. Antibiotics significantly reduced SCFAs concentrations compared with the C-VPA (*p* = 0.001), P-VPA (*p* = 0.042), ABx + probiotic (*p* = 0.013), probiotic (*p* = 0.002) and control (*p* = 0.025) groups (Table 2). Additionally, acetic acid (*p* = 0.001), butyric acid (*p* = 0.001) and propionic acid (*p* = 0.001) concentrations were also diminished within the ABx group (Fig. 4). Associated to the probiotic treatment, SCFAs concentrations did not change significantly in case of the majority of groups (Table 2). However, for the ABx + probiotic treated animals, significant decrease (*p* = 0.001) was identified after the usage of antibiotics. It is worth noting that the probiotic treatment was able to restore this decrease to the original state (Fig. 5), but there were no differences before and after both of the treatments in the SCFAs concentrations. Although, there were no significant changes in the butyric acid concentrations, there was a tendency for higher butyrate composition in the ABx + probiotic rats compared to animals of the other groups. Examination of the prenatal VPA exposed rats did not present significant differences among and within these animal groups.

**Table 2:** Analysis of the short chain fatty acids (acetic acid, propionic acid and butyric acid) concentrations (100 mg/mmol/l) in the faecal samples before and after the treatments.

SCFA concentrations (100 mg/mmol/l)	ABx n = 8	ABx + probiotic n = 8	Probiotic n = 8	P-VPA n = 7	C-VPA n = 7	Control n = 7
**Before treatments**						
Acetic acid	6.25 (4.03–8.54)	5.50 (3.44–7.09)	4.75 (4.04–5.64)	8.79 (7.38–9.02)	7.16 (6.36–7.78)	7.13 (4.73–8.25)
Propionic acid	2.59 (1.88–3.02)	1.91 (1.38–2.33)	1.70 (1.47–2.44)	3.24 (2.78–3.79)	2.49 (2.09–2.92)	1.80 (1.63–3.14)
Butyric acid	1.36 (1.18–1.45)	1.00 (0.93–1.20)	0.93 (0.86–1.15)	1.92 (1.39–2.16)	1.48 (1.14–2.10)	1.10 (0.99–1.59)
Total	9.76 (7.56–13.63)	9.50 (5.80–10.37)	7.61 (6.69–9.16)	14.22 (11.24–14.83)	10.86 (10.05–12.47)	10.67 (7.58–13.04)
**After treatments**						
Acetic acid	1.64 (0.00–2.90)^abcde^	4.03 (2.37–5.11)^a^	4.14 (2.97–7.14)^b^	5.56 (4.49–8.60)^c^	7.77 (6.97–8.25)^d^	4.67 (2.85–5.28)^e^
Propionic acid	0.64 (0.00–1.02)^abcde^	1.30 (0.74–1.54)^a^	2.45 (1.48–2.57)^b^	2.82 (2.35–3.40)^c^	2.98 (2.60–3.50)^d^	2.09 (1.57–2.40)^e^
Butyric acid	0.36 (0.00–0.54)^abcde^	1.30 (1.19–1.62)^a^	0.96 (0.75–1.23)^b^	1.15 (0.76–2.22)^c^	2.23 (1.77–2.33)^d^	1.22 (1.00–1.35)^e^
Total	2.65 (0.00–4.44)^abcde^	6.46 (4.18–8.50)^a^	7.40 (5.82–10.58)^b^	9.14 (7.79–14.22)^c^	13.30 (11.44–13.56)^d^	7.79 (5.73–8.62)^e^

ABx: broad-spectrum antibiotics treated group, ABx + probiotic: broad-spectrum antibiotics- and probiotic treated group, Probiotic: probiotic treated group, P-VPA: valproic acid and probiotic treated group, C-VPA: valproic acid treated group, Control: control group. Values of the concentrations are median (IQR). Kruskal–Wallis test, Mann–Whitney *U*-test and Friedman test. Between the groups significances (*p* < 0.05) are represented by distinct (a–e) lowercase letters.

### Histology

At the end of behavioral experiments, after preparation of the brains, the diameter of the major hippocampal regions (subiculum, CA1, CA2, CA3, dentate gyrus) was compared among 6–6 animals/groups. Based on the results, diameter of the various hippocampal regions demonstrated extreme differences (Supplementary Table S2; Supplementary Fig. S2), nevertheless, the thickness of each region also revealed remarkable alterations within the groups. Therefore, our histological data, at their present form, proved not to be correctly interpretable.

## Discussion

The purpose of this study was to assess the impact of broad-spectrum antibiotics treatment on social behavior in adulthood. To our best knowledge, this report is the first one to demonstrate that chronic depletion of the gut microbiota in adulthood induces profoundly similar social behavioral abnormalities to those observed in animals of the VPA rat model of ASD. Furthermore, as it was supposed, after the antibiotics treatment, our PM was able to re-establish the normal social behavior. In addition to that our PM treatment was capable to markedly reduce the social abnormalities in the VPA animal model, it also appears that even distinct changes of the microbiome could result in remarkable changes in a completely developed brain. According to our present findings, these changes take place in a non-SCFA dependent way.

The instantaneous effect that the GI microbiome exerts on the social behavior has been studied primarily with germ free animals (total lack of microbes) and with certain antibiotic treatments on rodents in their early pre-or post-natal period of life^33^. These studies primarily revealed impairments of sociability. Furthermore, colonizing germ free mice with normal faecal microbiota was able to restore the sociability defects. Based on these results, it is reasonable to propose that the gut microbiota is involved in integratory processes of social development^48,49^. Despite all these facts the effect of broad-spectrum antibiotics treatment on social behavior is less studied in adulthood. One of the fundamental findings of the current study highlights that the antibiotics induced bacterial depletion in adulthood can elicit the same type deficits of the social interaction as those observed in the VPA autism rat model. The sociability index results, indicating a preference to the stranger rat, presented that rats of the C-VPA- and ABx groups display social interaction deficits. These findings suggest that chronic broad-spectrum antibiotics treatment in adulthood negatively affects the social behavior, moreover, it seems as if these deficits were the same type as those we could identify in the VPA rat model of ASD. This finding appears to support the notion that the antibiotics-modified microbiome can act as a causal agent and a risk factor in the development of ASD^27,30,50^. Despite these social abnormalities, group differences were not found in the total distance travelled and in the latencies to explore the stranger cage. Otherwise, a pilot experiment with LiCl induced visceral illness was not able to result in reduced sociability what we detected in the microbiome depleted ABx rats. These results suggest that impaired sociability is likely cannot be due to the general consequence of visceral discomfort or pain in these animals. The above data underline the importance of disruption of the healthy balance of microbial community and its specific impact on the microbiome–gut–brain axis that leads to the deficits of social behavior regardless of whether visceral discomfort exists or not.

It is established that antibiotics- or VPA treatments interfere with the physiology of the animals disparate ways. In the former case, antibiotics administration strongly depletes gut microbiota, and thus, triggers alterations of the microbiome–gut–brain axis that ultimately lead to behavioral (as well as molecular) deviations^34,51^. In the latter case, prenatal VPA exposure was shown to modify histone deacetylase activity, to alter gamma-aminobutyric acid (GABA) or Wnt (wingless‐type) signaling, and/or to disturb axonal remodeling in the developing neurons^20,23,52^. These mechanisms can provoke dysfunctions in several brain areas, generating morphological changes, especially in cortical and hippocampal regions^20,53^. Nevertheless, our histological analysis revealed very high variability within and among the animals, hence, we could not verify that such differences (e.g. in the thickness of hippocampal regions) indeed exist between the VPA treated and other groups. Even though the treatments appeared to act divergent ways, we still noticed similar social behavioral alterations between these groups. Therefore, to more precisely explore the role of the GI microbiome in the development of ASD symptoms, we approached this issue from another direction and introduced a probiotic therapy to interfere these treatments. Previous studies have shown that application of certain probiotics have beneficial effects on antibiotics-induced physiological and psychological abnormalities^54,55^. Moreover, as different gut microbial community was found in ASD patients in contrast to the healthy individuals, researchers attempted to modify the gut microbiome via probiotics and some results indicate beneficial effects on both behavioral and GI manifestations of ASD^56–59^. In addition, VPA rat model not just imitate ASD symptoms, it also has a transgenerational impact on the gut microbiota^60–62^. Nevertheless, limited research is available where probiotics are investigated in the VPA animal model as they could be effective therapy, only one study has recently revealed that VPA induced behavioral alterations could be reduced by daily supplementation with Lactobacillus strains^63^. In our present study, it is demonstrated for the first time that specified PM can be a potential novel approach to improve social behavioral alterations both in the VPA- or antibiotics induced animal model. Our PM was able to improve the preference to the stranger rat in both of the C-VPA and ABx treated groups, thus antisocial behavior was reduced. Moreover, the present results demonstrated that the same behavior can be seen in the C-VPA and ABx treated groups as it appeared in the control group. However, the PM itself was not able to significantly change all aspects of the behavior, only in the frequency of the social zone exploration was detected notable difference between the probiotic and the control rats, but this was presumably generated by the fact that probiotic treated animals spent more time in the social zone once they entered there.

Despite the fact that both models developed by different mechanisms, quite similar social behavioral abnormalities were noticed, additionally, our PM was able to reconstruct these behavioral phenomena just as they appear in the control rats. Regarding these consequences, it is suggested that in both models the protective effects of the probiotic treatment get exerted in the same way. A series of studies have described that SCFAs improve the gut health, regulate immune mechanisms and they may possess neuroactive properties^43,64–67^. However, findings from ASD human studies on the associations among the three main SCFAs have proved to be divergent^68–70^. At the same time, in rodent models, the administration of propionic acid could produce behavioral changes closely resembling those found in ASD^71^, and in the VPA autism model changes of the SCFAs concentrations were also observed^60^. In spite of all these, the analysis of the main SCFAs did not show significant differences between the VPA treated and the control animals, and, after the probiotic therapy, there were also no remarkable effects seen in the SCFAs productions. Nevertheless, it is clear that the antibiotics treatment itself significantly decreases the levels of all the examined SCFAs, referring to the highly decreased total amount of the microbiome in these animals. In spite of the fact that the PM considerably elevated the concentration of the SCFAs after the antibiotics treatment, we did not reveal extreme alterations among the groups after the end of the treatments. However, it is reasonable to suppose that the probiotic impact would be necessarily stronger after the antibiotics administration than in case of challenging the compact, untreated microbiome community. The present results undeniably indicate that the change of concentration of the main SCFAs cannot be the sole causal factor that determines how the PM exerts its positive impact on the social behavior.

Increasing amount of data support the consideration that the manifested inflammation and metabolic patterns are quite comparable in both investigated animal models^72,73^. While, in the antibiotics-treated rat model, the inflammatory processes and the serotonergic system appear to be linked to the gut dysbiosis^34,74,75^, in the VPA animal model the elevated pro-inflammatory state, chronic glial activation and disturbance of the serotonergic system are caused by impact of the VPA to the developing brain^75–78^. Therefore, these observations encourage us to maintain the presumption that our PM made an effect on the serotonergic system without the mediation of the alterations of the SCFAs, thus, providing us the opportunity to hypothesize this to be the common way how the probiotic formulation can re-establish the behavioral alterations. It could occur in a way that modification and reduction of the inflammatory processes (diminishing the gut permeability) altogether with the altered microbiome could interact and alter the serotonergic system^79–83^. Since GI microbiota can directly or indirectly influence the tryptophan availability and the serotonin synthesis, thus, it ultimately influences the regulation of the kynurenine pathway. This pathway controls the production of the neuroprotective kynurenic acid (*N*-methyl-d-aspartate (NMDA) receptor antagonist) and that of the neurotoxic quinolinic acid (NMDA receptor agonist)^82^. So, the modulations of this system could lead to altered expression levels of NMDA^82,84,85^. It has been demonstrated that antibiotics could also alter NMDA receptor subunit expressions^86^. Recent studies have also identified that post-natal VPA treatment enhanced NMDA receptor functioning in the brain which may indicate a compensatory homeostasis with the presence of an excitatory/inhibitory imbalance during development. In subsequent experiments, when using pharmacological suppression therapy or NMDA receptor antagonists, they were able to normalize social deficits^60,76,87,88^. These results highlight the impact of these transcriptional modifications which could be the last elements in the way how our probiotic could restore the antisocial behavior, since NMDA receptors play an essential role in complex cognitive and social behavioral processes^89,90^. Furthermore, *Lactobacillus* spp. and *Bifidobacterium* spp. as GABA-producing species may be able to good candidates to theoretically change glutamate/GABA ratio thus it could be a promising strategy to treat ASD associated social behavioral symptoms^91,92^. Nevertheless, further studies are needed to measure constituent elements of inflammatory processes, metabolic patterns and NMDA receptor expression levels in both animal models along with measuring these values after the probiotic treatment. Furthermore, additional investigations are necessary to examine changes of other minor SCFAs, as well as to apply other behavioral tests to explore wider ranges of behavioral alterations. Indeed, future studies should also clarify whether the 2 weeks of our PM exposure ensures only shorter, temporary or lasting effects on the social behavior. Furthermore, future studies are expected to reinforce the therapeutic efficacy of our PM on the ASD.

Taken together, our data confirm that broad-spectrum antibiotics treatment during adulthood can induce antisocial behavior similar to that observed in the VPA autism animal model. The current study suggests that the homeostatic balance of the GI microbiome has a profound effect on the social behavior. To our best knowledge this study is among the first ones to demonstrate that specific probiotic mixture can restore the same type of antisocial behavioral phenomena in these two disparate animal models, developed by distinct mechanisms. Based on the present data, this probiotic formulation targets a common pathway with a non-SCFA dependent manner. Overall, this study provides preliminary evidence for that GI microbiome, more specifically some bacterial combinations, appears to have therapeutic value to cure or at least attenuate the condition of social behavior, and thus, to get prepared to act as a proper therapeutic agent to eliminate symptoms of antisocial behavior in the ASD.

## Supplementary Information

Supplementary Information 1.

Supplementary Information 2.

## Data Availability

All data generated or analysed during this study are included in this published article [and its supplementary information files].
