# Supplementary material for: Novel probiotic treatment of autism spectrum disorder associated social behavioral symptoms in two rodent models
Source: Sci Rep. 2022 Mar 30;12:5399. doi: 10.1038/s41598-022-09350-2 (PMC8967893; doi:10.1038/s41598-022-09350-2)
Supplement: Supplementary file 1 — Supplementary Information 1. [file 41598_2022_9350_MOESM1_ESM.pdf]

Novel probiotic treatment of autism spectrum disorder associated social behavioral symptoms in two rodent models

Kitti Mintál[1,2*], Attila Tóth[1,2], Edina Hormay[1,2], Anita Kovács[1], Kristóf László[1], Anita Bufa[3], Tamás Marosvölgyi[3], Béla Kocsis[4], Adorján Varga[4], Zoltán Vizvári[2,5], Renáta Cserjési[6], László Péczely[1], Tamás Ollmann[1], László Lénárd[1,2], Zoltán Karádi[1,2]

1 Institute of Physiology, Medical School, University of Pécs, Pécs, Hungary

2 Cellular Bioimpedance Research Group, Szentágothai Research Centre, University of Pécs, Pécs, Hungary

3 Institute of Bioanalysis, Medical School, University of Pécs, Pécs, Hungary

4 Department of Medical Microbiology and Immunology, Medical School, University of Pécs, Pécs, Hungary

5 Department of Environmental Engineering, Faculty of Engineering and Information Technology, University of Pécs, Pécs, Hungary

6 Institute of Psychology, ELTE Eötvös Loránd University, Budapest, Hungary

* kitti.mintal@aok.pte.hu

Supplementary information:

Pilot experiment with lithium chloride

Lithium chloride (LiCl) is a well-known toxicous chemical which causes visceral illness, and in this pilot experiment it was used to generate intestinal discomfort. Rats were injected intraperitoneally with LiCl (125 mg/kg/ml) (1) and half hour after the injection three chamber social interaction test was conducted.

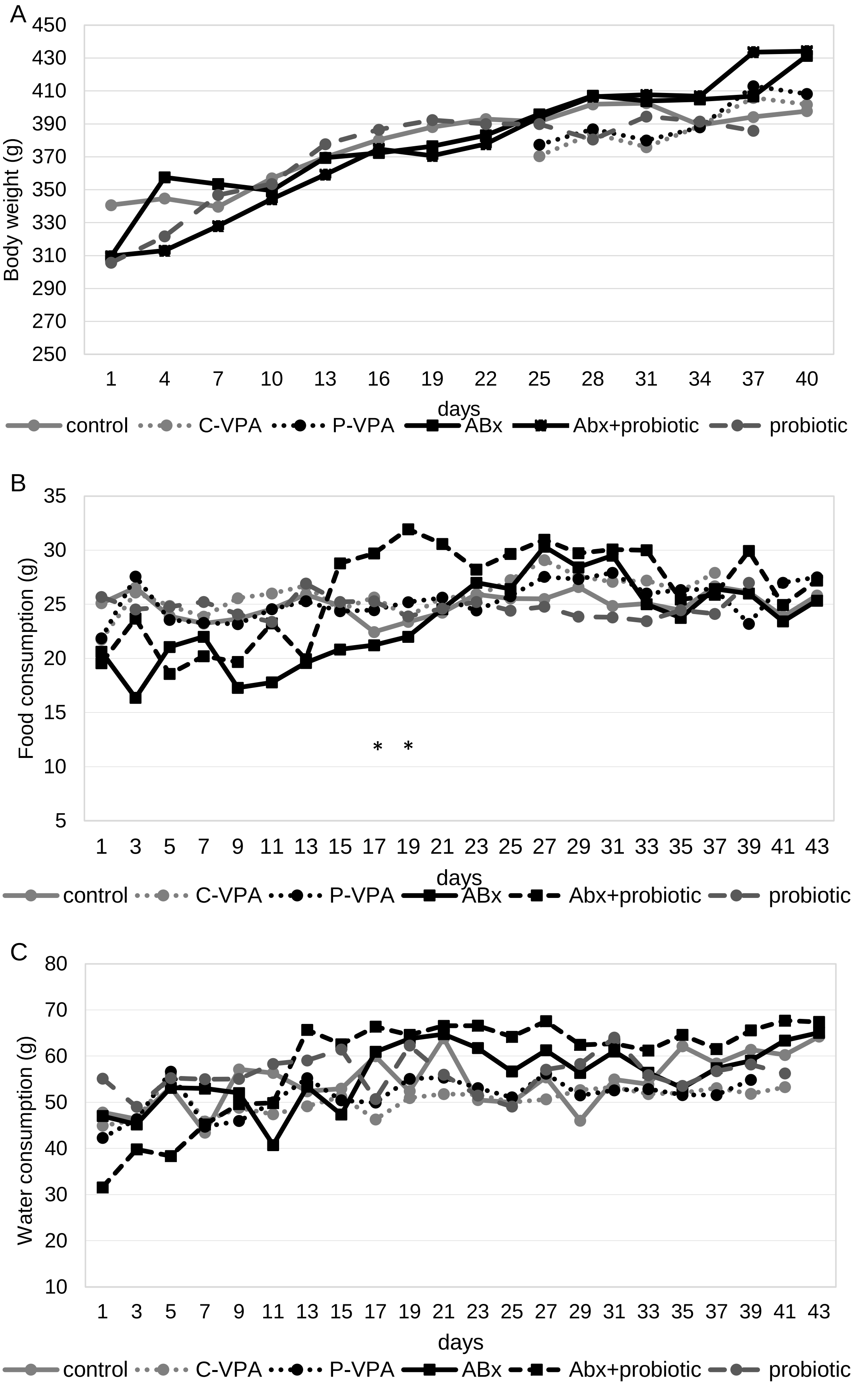
A
Body weight (g)
450
430
410
390
370
350
330
310
290
270
250
1 4 7 10 13 16 19 22 25 28 31 34 37 40
days
control C-VPA P-VPA ABx Abx+probiotic probiotic
B
Food consumption (g)
35
30
25
20
15
10
5
* *
1 3 5 7 9 11 13 15 17 19 21 23 25 27 29 31 33 35 37 39 41 43
days
control C-VPA P-VPA ABx Abx+probiotic probiotic
C
Water consumption (g)
80
70
60
50
40
30
20
10
1 3 5 7 9 11 13 15 17 19 21 23 25 27 29 31 33 35 37 39 41 43
days
control C-VPA P-VPA ABx Abx+probiotic probiotic

Supplementary Figure S1. Animals' body weights (A) food (B) and water (C) consumptions during the treatments. ABx: broad-spectrum antibiotics treated group, ABx+probiotic: broad-spectrum antibiotics- and probiotic treated group, Probiotic: probiotic treated group, P-VPA: valproic acid and probiotic treated group, C-VPA: valproic acid treated group, Control: control group without any treatment. One-way ANOVA (* p<0.05). Data graphed as mean.

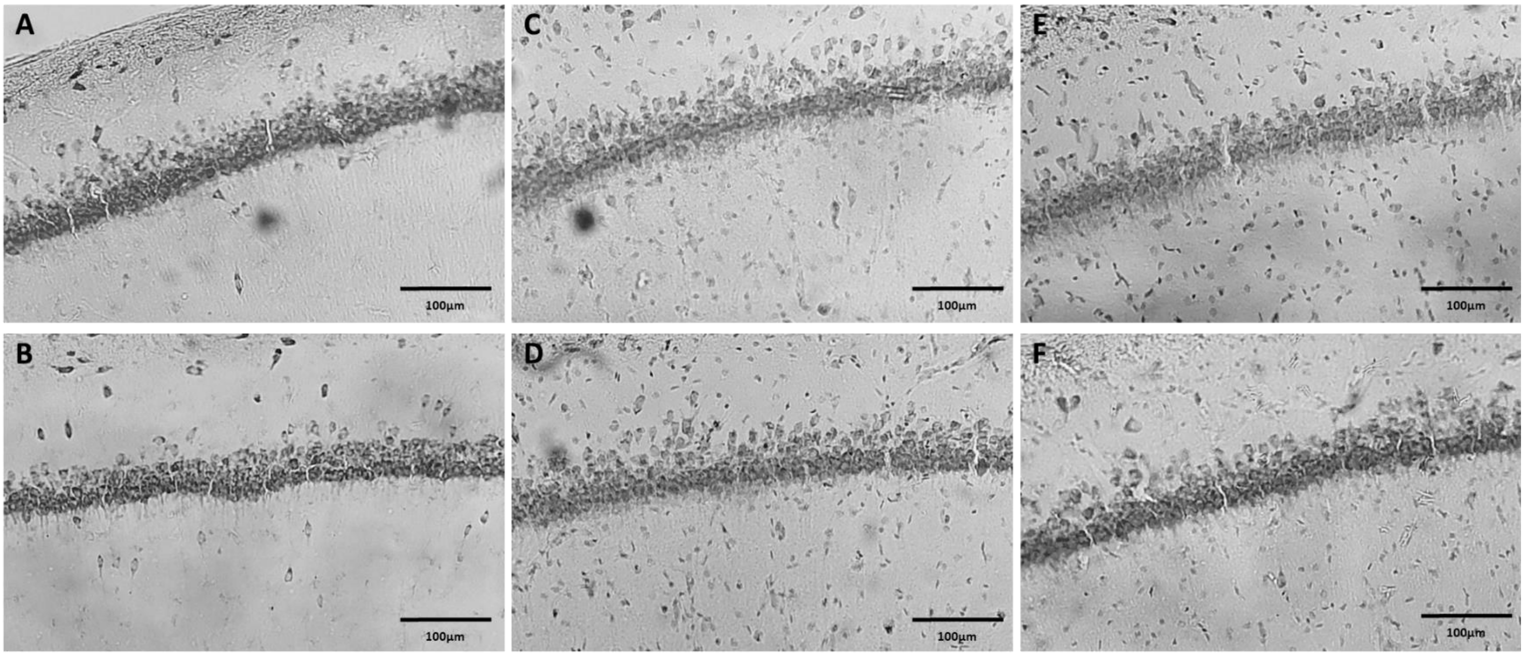

Supplementary Figure S2. Cresyl violet staining of the CA1 hippocampal layer. Photomicrographs of coronal sections of the hippocampal CA1 regions A: broad-spectrum antibiotics treated group, B: broad-spectrum antibiotics- and probiotic treated group, C: probiotic treated group, D: valproic acid and probiotic treated group, E: valproic acid treated group, F: control group. The scale bar for all of images is 100 μm.

3 chamber social interaction test	LiCl (n=8)
**Stranger cage latency (sec)**	23 (8.50-170.25)
**Empty cage latency (sec)**	59 (3.75-200.50)
**Sociability index**	0.13 (-0.47-0.84)
**Total distance moved (cm)**	1845.97 ± 86.64
**Time spent with the social zone exploration (sec)**	306.94 ± 82.93
**Time spent with the non-social zone exploration (sec)**	264.37 ± 86.84
**Frequency with the social zone exploration**	3.13 ± 1.04
**Frequency with the non-social zone exploration**	2.25 ± 0.75
**No. of interactions with the stranger rat**	16.13 ± 4.00
**No. of interactions empty cage**	6.50 ± 1.95

Supplementary Table S1. Three chamber social interaction test. LiCl: lithium chloride treated animals. The results did not show significant differences between the LiCl treated rats and rats of the other groups. Values of the stranger cage latency, the empty cage latency and sociability index are median (IQR) and the total distance moved, time spent with either the social- or non-social zone exploration, frequency with either the social- or non-social zone exploration and the number of interactions with the stranger or empty cage are means and SEMs. One-way ANOVA, Kruskal–Wallis test and Mann–Whitney U-test.

diameter of the hippocampal regions (µm)	ABx n=6	ABx+probiotic n=5	probiotic n=5	P-VPA n=5	C-VPA n=5	Control n=5
**subiculum**	45.36 (37.97-57.03)[a]	43.88 (37.20-51.77)[abcd]	46.06 (39.55-53.87)[be]	47.02 (40.35-55.83)[cf]	46.37 (38.97-54.17)[dg]	41.03 (33.69-48.64)[aefg]
**Ca1**	39.68 (34.71-43.61)[abcd]	35.15 (30.79-39.78)[abcde]	40.19 (35.00-45.89)[abcde]	44.46 (38.39-49.58)[ce]	43.45 (38.33-49.58)[de]	38.55 (33.54-43.49)[e]
**Ca2**	63.92 (57.17-71.07)[abcd]	62.63 (55.26-68.88)[efgh]	69.02 (56.34-77.79)[aeb]	71.78 (62.84-81.89)[bfj]	70.93 (60.82-80.51)[cgj]	67.64 (57.54-78.38)[dh]
**Ca3**	67.79 (56.32-84.70)[abc]	64.15 (53.54-80.03)[acd]	61.65 (48.31-81.22)[bedc]	68.64 (53.76-87.36)[df]	77.75 (65.34-92.98)[cf]	67.39 (52.44-81.29)[e]
**dentate gyrus**	65.89 (56.43-78.14)[a]	61.22 (53.19-71.94)[a]	63.73 (53.44-80.11)[a]	69.22 (59.83-80.74)[a]	65.90 (57.57-77.42)[a]	61.73 (53.23-72.36)[a]

Supplementary Table S2. Analysis of the diameter (µm) of the hippocampal regions (subiculum, CA1, CA2, CA3, dentate gyrus) ABx: broad-spectrum antibiotics treated group, ABx+probiotic: broad-spectrum antibiotics- and probiotic treated group, Probiotic: probiotic treated group, P-VPA: valproic acid and probiotic treated group, C-VPA: valproic acid treated group, Control: control group. Values of the diameters are median (IQR). Kruskal–Wallis test, Mann–Whitney U-test and Friedman test. Between the groups significances ($p<0.05$) are represented by distinct (a-j) lowercase letters.

References:

1. Buffalari DM, Mollica JK, Smith TT, Schassburger RL, Rinaman L, Thiels E, et al. (2016): Nicotine Enhances Footshock- and Lithium Chloride-Conditioned Place Avoidance in Male Rats. *Nicotine Tob Res*. 18:1920-1923.
